# Validity and interexaminer reliability of a new method to quantify skin neurofibromas of neurofibromatosis 1 using paper frames

**DOI:** 10.1186/s13023-014-0202-9

**Published:** 2014-12-05

**Authors:** Karin SG Cunha, Rafaela E Rozza-de-Menezes, Raquel M Andrade, Amy Theos, Ronir R Luiz, Bruce Korf, Mauro Geller

**Affiliations:** Postgraduate Program in Pathology, School of Medicine, Universidade Federal Fluminense, Rio de Janeiro, Brazil; Neurofibromatosis National Center (Centro Nacional de Neurofibromatose), Rio de Janeiro, Brazil; Children’s South Specialty Clinic, University of Alabama at Birmingham, Alabama, USA; Instituto de Estudos de Saúde Coletiva, Universidade Federal do Rio de Janeiro, Rio de Janeiro, Brazil; Department of Genetics, School of Medicine, University of Alabama at Birmingham, Alabama, USA; Department of Immunology and Microbiology, School of Medicine, Centro Universitário Serra dos Órgãos, Rio de Janeiro, Brazil; Instituto de Puericultura e Pediatria Martagão Gesteira, School of Medicine, Universidade Federal do Rio de Janeiro, Rio de Janeiro, Brazil

**Keywords:** Neurofibromatosis 1, Neurofibroma, Reliability and validity, Validation studies

## Abstract

**Background:**

Skin neurofibromas represent one of the main clinical manifestations of neurofibromatosis 1, and their number varies greatly between individuals. Quantifying their number is an important step in the methodology of many clinical studies, but counting neurofibromas one by one in individuals with thousands of tumors is arduous, time-consuming, and subject to intra and interexaminer variability. We aimed to evaluate the efficacy of a new methodology for skin neurofibromas quantification using paper frames.

**Methods:**

The sample comprised 92 individuals with NF1. Paper frames, with a central square measuring 100 cm^2^, were placed on the back, abdomen and thigh. Images were taken, transferred to a computer and two independent examiners counted the neurofibromas. The average number of neurofibromas/100 cm^2^ of skin was obtained from the mean of the three values. The differences in the quantity of neurofibromas counted by two examiners were evaluated with Intraclass correlation coefficient (ICC), paired *t-*test, Bland-Altman and survival-agreement plots. To evaluate the predictive value of the method in obtaining the total number of neurofibromas, 49 participants also had their tumors counted one by one. Reproducibility was assessed with Pearson’s correlation coefficients and simple linear regression model.

**Results:**

There was excellent agreement between examiners (ICC range 0.992-0.997) and the total number of skin neurofibromas could be predicted by the adhesive frames technique using a specific formula (*P* < 0.0001).

**Conclusions:**

In this article we describe a reliable, easy and rapid technique using paper frames to quantify skin neurofibromas that accurately predicts the total number of these tumors in patients with NF1. This method may be a useful tool in clinical practice and clinical research to help achieve an accurate quantitative phenotype of NF1.

**Electronic supplementary material:**

The online version of this article (doi:10.1186/s13023-014-0202-9) contains supplementary material, which is available to authorized users.

## Background

Neurofibromatosis 1 (NF1, OMIM 162200) is a common autosomal dominant genetic disorder caused by mutations in the *NF1* gene. It presents extremely variable expressivity and can cause diverse clinical manifestations, including café-au-lait spots, axillary and inguinal freckling, Lisch nodules, and multiple benign peripheral nerve sheath tumors called neurofibromas [1,2]. Neurofibromas occur mainly in cutaneous and subcutaneous tissues, usually begin to appear during puberty and their number tends to increase progressively with age [1,3]. To date, although drug trials have been initiated looking for medications that halt or slow the growth of neurofibromas, no beneficial therapy is known.

The number of skin neurofibromas varies widely between individuals (from a few to more than thousands), even between individuals from the same family. Currently, only two genotype-phenotype correlations are well established: individuals with deletion of the entire *NF1* gene tend to have a large number and early development of skin neurofibromas; those with a 3-base pairs in-frame deletion in exon 17 of the *NF1* gene, do not develop cutaneous neurofibromas [4-7]. It has been suggested that other factors may contribute to this variable expression, such as hormones, epigenetic alterations, as well as modifier genes not related to the *NF1* locus itself [8-11].

Quantifying the number of neurofibromas is important for genotype-phenotype studies [12] and also for future clinical trials of medications intended to treat skin neurofibromas. Counting neurofibromas one by one in individuals with NF1 who may have thousands of tumors is arduous, time-consuming, and subject to intra- and interexaminer variability. In many studies, NF1 individuals are categorized according the number of neurofibromas, and some authors do not report if the categorization was performed based on counting neurofibromas one by one or if the exact number of tumors was not obtained and an estimative of their number was used, which of course is also subject to variability [3,12-16].

Our aim was to evaluate the efficacy of a new methodology for skin neurofibromas quantification using paper frames, investigating interexaminer reliability and value of the approach in predicting the total number of skin tumors.

## Methods

### Case selection

This study protocol was approved by the Review Board of the Antônio Pedro University Hospital of Universidade Federal Fluminense, Niterói, RJ, Brazil (#121/11). The individuals’ written, informed consent was obtained and the Declaration of Helsinki protocols were followed. A total of 92 post-pubertal NF1 individuals were included in this research. NF1 diagnosis was based on the clinical criteria of National Institutes of Health [2]. The exclusion criteria were: presence of a large plexiform neurofibroma on the back, thigh, or abdomen that crossed the midline; history of surgical removal of a large number of skin neurofibromas from back, thigh, or abdomen (more than ten within the five years prior to enrollment in the study); previous treatment with chemotherapy or radiotherapy, as well as with any drug (such as interferon, retinoic acid, thalidomide, or farnesyl transferase inhibitor) tested for efficacy in neurofibroma treatment.

### Study design

After signing the informed consent, study participants had their data collected: age, gender and skin color. Then, the study participants were clinically evaluated after getting undressed, keeping only their lower underwear.

### Quantification of neurofibromas using the paper frames method

**Figure 1:**
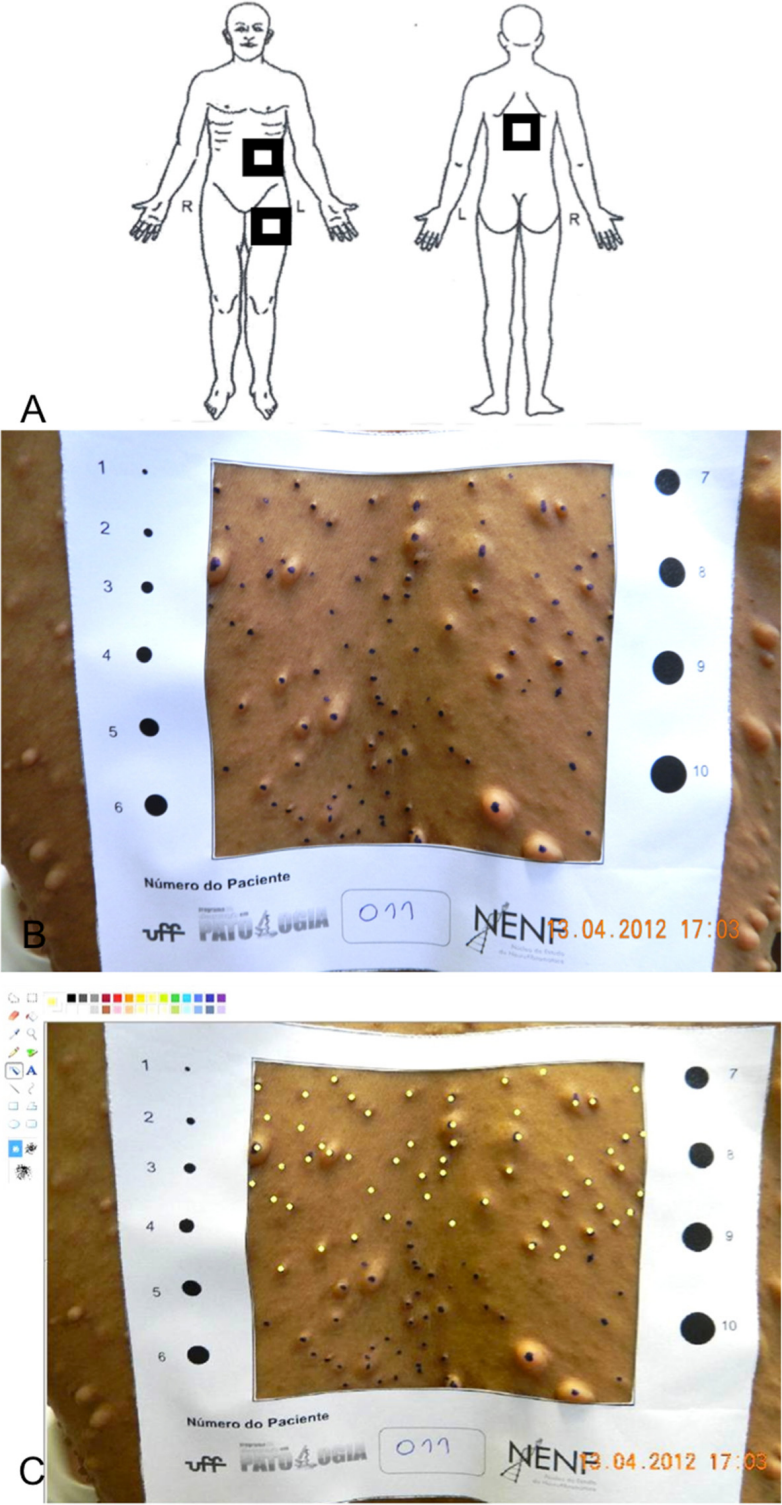
**Paper frames method for counting skin neurofibromas. (A)** Schematic image of paper frames attachment: An adhesive backing from paper frame was removed and it was placed on the individual’s abdomen with a bottom corner placed just left of the individual’s umbilicus, left thigh and back with the top corners put just under the individual’s scapula. If a large plexiform tumor existed on the left side of the abdomen or thigh, the opposite side would have chosen for paper frames attachment. **(B)** The paper frame in the back presenting skin neurofibromas marked by washable pen. **(C)** The process of skin neurofibromas counting using Paint® software (yellow points represent neurofibromas that had already been counted).

The quantification technique uses paper frames, with a central square measuring 100 cm^2^, which are placed in three specific areas of the body (back, abdomen and thigh) (Figure 1A). After placing the paper frames on the specific areas, two photographs were taken from each paper frame, using a digital camera (Sony Cyber-shot DSC-W30, Tokyo, Japan). Then, to facilitate the analysis of the photographs, a washable pen was used to mark the cutaneous neurofibromas, as well as the subcutaneous neurofibromas (with the aim to facilitate their distinction from café-au-lait spots). Two additional pictures were taken after marking the neurofibromas. The photographs were obtained with resolution of 300 dpi in JPEG format.

The images were transferred to a computer and the clearest photograph containing the neurofibromas marked with the washable pen was used to count the tumors (Figure 1B). All the pictures were analyzed using the software Paint® (Microsoft Corporation, Redmond, WA, USA). The examiner used the spray tool to highlight the skin neurofibromas that had already been counted, avoiding the possibility of counting the same neurofibroma twice (Figure 1C). The average number of skin neurofibromas per 100 cm^2^ of skin was defined as the mean of the three areas.

### Evaluation of interexaminer reliability of the paper frames method

To evaluate the interexaminer reliability of quantification of neurofibromas using the paper frames method, all the pictures were analyzed by two independent trained examiners: RMA (examiner A) and RERM (examiner B). The interexaminer analysis was calculated using the mean of the three counts (abdomen, back and thigh).

### Evaluation of paper frame method in predicting the total number of skin neurofibromas

To evaluate the predictive value of the paper frames method in obtaining the total number of skin neurofibromas, 49 participants of the study had their tumors counted one by one by a trained examiner (RMA; examiner A). Neurofibromas on genitals and scalp were not included.

### Statistical analysis

For statistical analysis, we used the SPSS software (IBM® SPSS®, v. 20.0, Armonk, NY, USA) and Excel 2011 (Microsoft Corporation, Redmond, WA, USA). *P* values less than 0.05 were considered statistically significant. Pearson’s correlation coefficient was used to evaluate the correlation between total number of skin tumors and age of NF1 patients. For this, patients were grouped into five categories of age: <20, 20–29, 30–39, 40–49 and ≥50 years old. For trend across ordering, the Mann-Whitney test was used to evaluate which age groups had significant difference in neurofibroma quantity.

The reliability of the paper frames method performed by the two independent examiners was analyzed by ICC, Bland-Altman plot associated with Spearman’s rho correlation coefficient and survival-agreement plot [17,18]. The paired t-test was also used to compare the means of the two examiners. For interexaminer reliability analyses, the results were also evaluated separately according to the skin color of the NF1 individuals and according to the average number of neurofibromas per 100 cm^2^ (<100 and ≥100 neurofibromas) with the aim to identify any fixed/proportional bias. The log rank test was applied in the survival-agreement plot to evaluate the difference in the quantity of neurofibromas obtained by both examiners according to skin color.

To estimate the inter-method reliability, Pearson’s correlation coefficient was used to evaluate whether the average number of neurofibromas in the paper frames corresponded to the estimated number of neurofibromas per 100 cm^2^ of skin based on the total number of neurofibromas. To obtain the estimated number of neurofibromas per 100 cm^2^ of skin based on the total number of neurofibromas, we used Mosteller’s formula to calculate the body surface area (BSA) [19]. BSA is defined as the square root of height (in centimeters) multiplied by weight (in kilograms) divided by 3,600.

The simple linear regression model was used to build an equation for predicting the approximate total number of skin neurofibromas from the paper frames method. The independent variable was assigned by exact total number of skin neurofibromas (y) and the dependent variable by paper frames method (x). This analysis was taken separately for each examiner. The R squared coefficient (R^2^) was used to verify the proportion of predictive values that were similar to the exact total number of tumors. Then, a simple linear regression model (y = α + βx) was used to convert the number of skin neurofibromas from the paper frame method into an exact total number of tumors [20].

## Results

Details of the clinical data and count of the skin neurofibromas of all individuals analyzed are shown in Additional file 1. Of the 92 individuals included in this study, 62 (67%) were females and 30 (33%) were males; 61 (66%) were white and 31 (35%) were black. The mean age was 40.6 (± 15.6) years (range 12–77 years). Of the 49 individuals who also had the total number of neurofibromas evaluated, 29 (59%) were females and 20 (41%) males; 26 (53%) were white and 23 (47%) were black. The mean age was 35.6 (± 15.2) years (range 12–75 years).

Regarding these 49 NF1 individuals who also had the total number of neurofibromas evaluated, the number of tumors strongly varied with age (r = 0.633, *P* < 0.0001); older individuals had higher number of neurofibromas. This variation of the number of neurofibromas according to age occurred until 39 years old (*P* ≤ 0.05). After 40 years old, no differences in neurofibroma number between ages were observed (*P* > 0.05). The total number of skin neurofibromas ranged from no tumors to 3,816 neurofibromas (485 ± 801; first quartile = 33, median = 183; third quartile = 515). Eighty-four percent of individuals had fewer than 1,000 neurofibromas and 16% had more than 1,000 neurofibromas; of which 4% had more than 3,000 tumors. The estimated number of neurofibromas per 100 cm^2^ of skin, based on the total number of neurofibromas, varied from 0 to 26.7 (2.9 ± 5.2, first quartile = 0.2, median = 1.1, third quartile = 3).

### Interexaminer reliability using paper frames method

There was no statistically significant difference when comparing the general means of the number of neurofibromas obtained from examiner A and examiner B (paired t-test; *P* = 0.236). The intraclass correlation coefficient (ICC) showed excellent homogeneity/reliability between the two examiners (Table 1).

**Table 1:** **Descriptive statistics and interexaminer reliability of number of neurofibromas using the paper frame’s method**

**Descriptive statistics**	**Examiner A**	**Examiner B**	**Differences (A-B)**
n	92	92	
Mean	67.09	66.17	0.93
Standard Deviation	76.79	72.67	7.47
Minimum	0	0	- 13
First Quartile	9.33	9.49	- 0.92
Median	38.49	38.99	0
Third Quartile	94.24	91.91	0
Maximum	348.66	330.66	42
Interexaminers			
ICC^1^			0.995 (0.992 – 0.997)
*P*-value (Paired *t* test)			0.236

^1^= Intraclass correlation coefficient (95% confidence interval) – two-way mixed ANOVA model, using the average of two measures (examiner A and examiner B).

**Figure 2:**
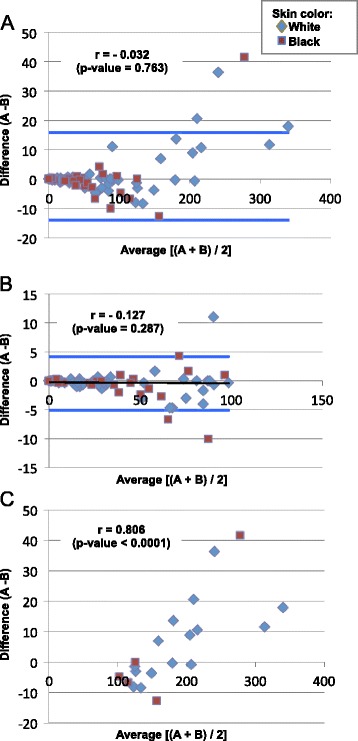
**Bland-Altman plot for the comparison between examiner A and examiner B using paper frames method. (A)** All sample of NF1 patients (n = 92); **(B)** Patients with < 100 neurofibromas from paper frames method (n = 72); **(C)** Patients with ≥ 100 neurofibromas from paper frames method (n = 20).

The results in Figure 2A show that, although the limits of agreement were obtained between both examiners, the examiner A tended to obtain higher values for the count of neurofibromas using the paper frames method. This discrepancy was more evident in individuals with more than 100 skin neurofibromas. Therefore, we decided to build Bland-Altman plots for two groups of participants: < 100 neurofibromas and ≥ 100 neurofibromas (Figure 2B and C, respectively). While in individuals with < 100 neurofibromas there was a fixed bias, counting errors in individuals with ≥ 100 neurofibromas tended to be higher as the number of skin neurofibromas increased (proportional bias). A strong positive correlation [20] between the differences and averages from both examiners were seen for individuals with more than 100 neurofibromas; thus the construction of the proposed limits by Bland and Altman were lost [17,21,22].

For individuals with < 100 neurofibromas, upper and lower limits of agreement were calculated: −0.46 (mean difference) ± 2 × 2.32 (standard deviation difference), resulting in an interval of −5.11 to 4.18. There was a similar distribution of white and black individuals, according to Bland and Altman plot (95% limits of agreement).

**Figure 3:**
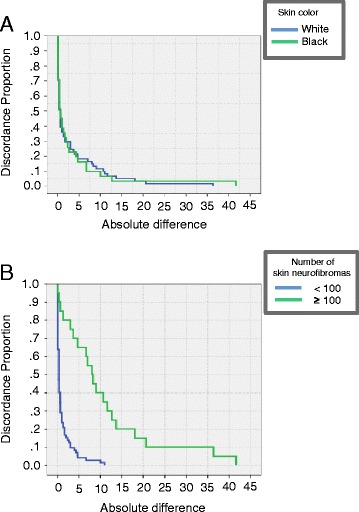
**Survival-agreement plot for examiner A and examiner B using paper frames method (n = 92). (A)** According to white (n = 61) and black (n = 31) skin color of patients with NF1; **(B)** According to the number of neurofibromas per 100 cm^2^ of skin: < 100 neurofibromas (n = 72) and ≥ 100 neurofibromas (n = 20).

The survival-agreement plot demonstrated agreement between the two examiners using the paper frames method [18]. In Figure 3A, when a threshold of 10 neurofibromas was seen, an agreement higher than 90% could be achieved. When the threshold of 20 neurofibromas was achieved, the agreement between A and B examiners was equal, being around 96%. As the difference of number of neurofibromas progressively increased, an agreement of almost 100% would be observed with a threshold of 36 neurofibromas in white individuals and 40 neurofibromas in black individuals (Figure 3A). Therefore, no important difference of agreement between the individuals’ skin color was observed (log rank test, *P* = 0.926).

Both examiners classified six individuals as having no skin neurofibromas, 66 (examiner A) and 65 (examiner B) with <100 neurofibromas and 20 (examiner A) and 21 (examiner B) with ≥100 neurofibromas, showing a similar distribution according to the second survival-agreement plot (Figure 3B). The agreement between examiners, using the paper frame method, was better for individuals with <100 neurofibromas (maximum threshold of 10 neurofibromas) than for individuals with ≥100 tumors (maximum threshold of 42 neurofibromas), but these differences did not have clinical importance since there was an agreement of approximately 99%.

### Predictive value of paper frames method of quantification of neurofibromas

**Figure 4:**
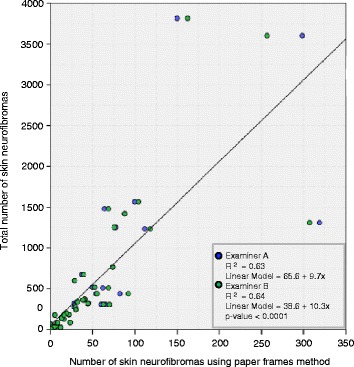
**Scatterplot of the total number of neurofibromas versus number of neurofibromas from paper frames method.** Examiner A: the regression line has the intercept α = 65.6 and the slope β = 9.7 (n = 49); Examiner B: the regression line has the intercept α = 38.6 and the slope β = 10.3 (n = 49). There is a statistically significant association (*P* < 0.0001) between the exact total number of skin neurofibromas and paper frames method for both examiners. Abbreviations: R^*2 =*^ coefficient of determination.

There was a statistically significant correlation (P <0.0001) between the average values (examiners A and examiner B) of skin neurofibromas from paper frames method and the estimated number of neurofibromas per 100 cm^2^ of skin, based on the total number of neurofibromas. The use of the linear regression model coefficients (α and β) predicted the values of Y [20]. Therefore, from a methodological point of view, the mean number of skin neurofibromas obtained from the paper frames corresponded to the total number of skin neurofibromas (*P* <0.0001), using the equation 65.6 + 9.7× for examiner A (R^2^ = 0.63) and the equation 38.6 + 10.3× for examiner B (R^2^ = 0.64) (Figure 4). The linear regression plot indicates a good predictive value for the paper frames method, having an excellent representativeness for the total number of skin neurofibromas.

## Discussion

We have demonstrated a new method to quantify skin neurofibromas in NF1 individuals using three paper frames with a 100 cm^2^ counting area. We showed that this method can predict the total number of skin neurofibromas and also presents a great interexaminer reliability because an acceptable agreement is assessed for ICC values ≥0.75; thus an excellent reproducibility is achieved for values close to +1 [23,24]. A very high ICC (0.995) value and a small interexaminer mean difference, which was less than one neurofibroma (−0.92), was observed. We measured the interexaminer reliability based on the differences between examiners, using ICC, which is a test that reflects the variability in mean score due to differences among examiners and how much variability is due to differences between examiners [25]. Although the Pearson’s correlation coefficient is frequently used to represent interexaminer reliability, this measure should be discouraged because it can be inconsistent [25].

Proportional bias could represent a practical problem of evaluating methods because one examiner may obtain consistently higher or lower number of neurofibromas than the other [17]. Bland-Altman and survival-agreement plots illustrate the information about possible fixed/proportional bias and degree of agreement/disagreement between examiners, respectively. We used these tests to investigate a possible proportional bias in individuals with ≥100 neurofibromas from the paper frames method because examiner A had a tendency to count fewer neurofibromas than examiner B in individuals with more than 100 tumors. Counting differences may occur due the superposition of the neurofibromas, which is commonly observed in areas with a high concentration of tumors, such as the torso [1]. Although this proportion could be confirmed by significant association between the differences and means of examiners on the Bland-Altman plot, this discordance (of up to 10 neurofibromas threshold) should be interpreted as a negligible value in obtaining the final number of skin neurofibromas, since there was a good agreement (approximately 99%) between both examiners using a survival-agreement plot [18]. In this study, we also evaluated whether black skin color could hinder the tumor counting. Our results showed that this variable was not a confounding factor, since it did not cause any proportional/fixed bias or significant difference in the number of tumors counted by the examiners.

The results using the frames to count neurofibromas show that this methodology may be useful for future studies with NF1 individuals. Although previous NF1 clinical studies and case reports, as well as case studies, have included neurofibroma quantification in their methods, the lack of an universal approach for quantification of these tumors is evident [3,12-16]. For example, in some studies, neurofibromas are counted by different examiners or the number of examiners that quantified the skin neurofibromas is not clear in the methodology [3,12]. In retrospective studies, as pointed by Duong et al. [3], the patients’ recruitment might also bias results, since skin neurofibromas would be assessed in clinical practice more accurately by dermatologists, for example [3]. Moreover, many authors do not mention which methodology was applied for neurofibroma quantification (counting one by one or an estimate number).

Quantifying the skin neurofibromas “one by one” may be very difficult and time consuming and is subject to intra- and interexaminer variation, mainly in individuals with more than 500 tumors. Therefore, researchers and clinicians commonly do not perform an exact count of the skin neurofibromas. Usually, individuals are categorized according to previously established count ranges, based on an estimated number of neurofibromas achieved by the overall clinical appearance. There are limitations when the number of tumors is predicted only by the overall clinical appearance. Individuals with many but small skin neurofibromas may have their total number of tumors underestimated. In many of these studies, for statistical analysis, patients are categorized in low and high tumor number groups. In the literature, there is a great variability in the cut-off values for the categorization of the NF1 individuals based on the number of neurofibromas. For example, the minimum value of the higher count range of neurofibromas in previous studies varies from ≥100, > 500 to >1,000 tumors [3,12,14,15]. Taking into consideration our NF1 sample, the number of neurofibromas ranged from 0 to 3,816 tumors, showing a tremendous variation. Evidently, the cut-off values of the count range of neurofibromas to create a categorization have important influence on the results. What are the optimal cut-off values for categorization? Future studies are necessary to perform this investigation and the use of the paper frames method may help in this research.

Our study confirms previously published data on skin neurofibromas, indicating an increase in number with age [3,26,27]. This is an important issue that should be taken into consideration when evaluating the number of skin tumors in NF1 individuals of different ages. For example, having more than 50 neurofibromas is rare in young adults and can be considered a high number of tumors, but having up to 50 neurofibromas can be considered a low number of tumors in older adults. Therefore, categorizing patients according to the number of neurofibromas for statistical analysis should take into consideration the age of the patients.

Individuals with NF1 present great variability not only in number of neurofibromas, but also in size of these tumors. To our knowledge, this variability in size of neurofibromas has never been evaluated in previous studies and it would be interesting to be included in future research. One of the advantages of paper frames method is the possibility of its usage, not only to evaluate the number, but also the size of the neurofibromas. It is possible because they present circles with different diameters (in millimeters) on the lateral margins. The paper frames method can be used to perform longitudinal studies, describing the evolution for each patient, regarding the number and the size of neurofibromas, over time. This longitudinal investigation of the number and size of neurofibromas would be of particular interest in drug tests.

In this study, we evaluated the cutaneous and subcutaneous neurofibromas together, but the number of these tumors can be achieved separately using the paper frame method, depending on the aims of the study. The presence of subcutaneous neurofibromas could be an expression of a more aggressive disease [28,29]. An association between malignant peripheral nerve sheath tumors and internal neurofibromas has been reported [29] and the presence of internal neurofibromas has recently been associated with at least 2 subcutaneous neurofibromas [16].

## Conclusions

In this article we describe a reliable, easy and rapid technique using paper frames to quantify skin neurofibromas that accurately predicts the total number of these tumors in patients with NF1. This method may be a useful tool in clinical practice and clinical research to help achieve an accurate quantitative phenotype of NF1. We are now working on the automation of the skin neurofibromas quantification using paper frames by pictures computerized analysis.

## Availability of supporting data

The data sets supporting the results of this article are included within the article and its additional file.

## Additional file

Additional file 1:
**Details of the clinical data and count of the skin neurofibromas of all individuals analyzed.**

## Abbreviations

NF1: Neurofibromatosis 1
ICC: Intraclass correlation coefficient
