# Supplementary material for: Validity and interexaminer reliability of a new method to quantify skin neurofibromas of neurofibromatosis 1 using paper frames
Source: Orphanet J Rare Dis. 2014 Dec 5;9:202. doi: 10.1186/s13023-014-0202-9 (PMC4267434; doi:10.1186/s13023-014-0202-9)
Supplement: Additional file 1: — Details of the clinical data and count of the skin neurofibromas of all individuals analyzed. [file 13023_2014_202_MOESM1_ESM.pdf]

Additional file 1 - Details of the clinical data and count of the skin neurofibromas of all individuals analyzed.

Patient Code	Age	Gender	Skin color	Total number of skin neurofibromas	Number of skin neurofibromas using the paper frames method - Examiner A				Number of skin neurofibromas using the paper frames method - Examiner B			
					Abdomen	Back	Tigh	Mean	Abdomen	Back	Tigh	Mean
1	46	F	W	NE	27	16	6	16,33	35	11	6	17,33
2	45	M	W	NE	142	202	13	119	115	124	13	127
3	66	F	W	NE	207	527	42	258,66	190	435	42	222,33
4	35	F	W	NE	26	48	8	27,33	24	48	8	26,66
5	71	F	W	NE	159	123	5	95,66	139	111	4	84,66
6	23	F	W	NE	124	121	16	87	124	121	16	87
7	52	F	W	NE	178	78	14	90	179	79	14	90,66
8	46	F	W	NE	227	319	16	187,33	149	356	16	173,66
9	35	M	B	NE	130	145	15	96,66	132	139	16	95,66
10	28	F	B	375	52	55	16	41	54	52	16	40,66
11	43	M	B	1254	113	101	18	77,33	109	101	17	75,66
12	51	M	W	NE	218	140	16	124,66	208	158	17	127,66
13	44	F	W	276	74	5	6	28,33	76	5	8	29,66
14	54	F	W	NE	111	90	20	73,66	114	95	21	76,66
15	67	F	W	NE	153	167	52	124	159	166	51	125,33
16	37	F	B	73	9	15	0	8	8	15	2	8,33
17	58	F	W	34	0	5	0	1,66	0	5	0	1,66
18	31	F	W	NE	29	19	5	17,66	29	19	5	17,66
19	26	F	W	520	47	87	15	49,66	47	98	13	52,66
20	24	F	W	9	2	1	1	1,33	2	1	1	1,33
21	49	F	W	NE	341	587	118	348,66	341	533	118	330,66
22	40	F	W	NE	35	9	7	17	35	7	8	16,66
23	56	F	B	3602	331	481	83	298,33	276	412	82	256,66
24	45	F	W	NE	251	314	61	208,66	241	294	64	199,66
25	55	F	W	NE	302	169	16	162,33	280	168	18	155,33
26	51	M	W	NE	243	184	15	147,33	251	188	14	151
27	55	F	W	NE	421	220	23	221,33	401	204	27	210,66
28	66	M	B	NE	167	189	19	125	170	186	19	125
29	54	F	W	NE	271	215	51	179	269	220	49	179,33
30	44	F	W	NE	66	66	45	59	74	57	41	57,33
31	37	F	W	769	86	116	20	74	86	115	20	73,66
32	23	F	W	27	4	11	22	12,33	3	11	22	12
33	32	F	W	40	3	7	2	4	3	7	2	4
34	31	M	W	NE	3	0	1	1,33	3	0	1	1,33
35	39	F	W	NE	289	342	30	220,33	313	253	33	199,66
36	37	F	B	364	37	74	8	39,66	37	71	8	38,66
37	16	M	B	35	6	9	0	5	7	9	0	5,33
38	13	F	W	6	1	1	1	1	1	1	1	1
39	37	M	W	1310	496	420	40	318,66	475	406	40	307
40	42	F	W	129	31	11	6	16	32	10	6	16
41	64	F	W	NE	92	49	15	52	91	51	15	52,33
42	47	M	B	NE	44	31	3	26	44	31	3	26
43	45	F	W	NE	7	9	0	5,33	8	9	0	5,66
44	19	M	W	14	0	2	0	0,66	2	0	0	0,66
45	68	M	W	25	3	4	1	2,66	4	3	1	2,66
46	43	M	W	41	5	13	2	6,66	5	13	2	6,66
47	45	F	W	NE	281	327	11	206,33	287	323	11	207
48	61	F	W	NE	154	88	1	81	156	86	1	81
49	25	F	B	183	40	22	3	21,66	41	22	3	22
50	54	M	B	600	48	35	3	28,66	49	35	2	28,66
51	56	F	W	NE	191	104	0	98,33	194	102	0	98,66
52	24	F	W	NE	17	71	5	31	18	72	5	31,66
53	29	F	W	140	24	14	1	13	26	14	1	13,66
55	26	F	B	12	0	0	0	0	0	0	0	0
56	39	F	B	NE	67	136	18	73,66	67	123	18	69,33
57	62	F	W	NE	152	196	40	129,33	167	204	42	137,66
58	77	F	B	NE	76	71	0	49	80	74	0	51,33
59	23	M	W	NE	53	27	1	27	53	29	1	27,66
60	41	F	W	200	38	15	2	18,33	40	15	2	19
61	28	F	W	NE	51	45	6	34	48	46	6	33,33
62	16	M	B	NE	0	0	1	0,33	0	0	1	0,33
63	48	F	W	NE	121	121	6	82,66	113	140	7	86,66
64	15	F	B	0	0	0	0	0	0	0	0	0
65	30	M	B	NE	5	7	1	4,33	4	7	1	4
66	37	F	B	NE	43	58	5	35,33	44	59	3	36
67	22	M	W	3	0	0	0	0	0	0	0	0
70	48	F	W	NE	37	5	2	14,66	39	5	3	15,66
71	17	F	W	32	7	7	0	4,66	7	7	0	4,66
72	54	F	W	NE	38	32	3	24,33	37	33	3	24,33
73	41	F	W	340	61	16	19	32	61	17	19	32,33
74	27	F	W	84	9	16	0	8,33	9	17	0	8,66
75	15	F	B	2	0	0	0	0	0	0	0	0
76	21	M	B	68	0	0	0	0	0	0	0	0
77	32	F	W	NE	109	121	21	83,66	108	127	21	85,33
78	13	F	B	2	0	0	0	0	0	0	0	0
79	33	F	B	87	24	42	3	23	24	43	4	23,66
80	46	M	B	320	73	59	1	44,33	74	58	2	44,66
82	39	M	W	308	79	113	4	65,33	123	83	4	70
83	47	M	W	1420	125	126	13	88	129	121	14	88
84	45	F	B	440	71	158	18	82,33	78	181	18	92,33
85	24	M	W	244	16	69	5	30	17	69	5	30,33
86	12	F	W	3	1	2	0	1	1	2	0	1
87	34	M	B	511	55	123	8	62	58	140	8	68,66
88	39	M	B	676	41	70	1	37,33	41	76	1	39,33
89	53	F	B	440	55	79	28	54	56	84	26	55,33
90	41	M	W	1483	116	68	8	64	111	87	8	68,66
91	72	M	B	1235	151	142	42	111,67	166	146	43	118,33
93	41	F	B	3816	205	222	23	150	238	228	22	162,66
94	29	M	B	310	130	48	3	60,33	138	48	3	63
95	75	M	W	178	10	3	3	5,33	10	4	2	5,33
96	39	M	W	178	40	5	2	15,66	38	8	2	16
97	56	F	B	1568	80	216	3	99,66	87	225	1	104,33

NE = not evaluated; F = female; M = male; W = white; B = black
